# Biogas Production from Citrus Waste by Membrane Bioreactor

**DOI:** 10.3390/membranes4030596

**Published:** 2014-08-27

**Authors:** Rachma Wikandari, Ria Millati, Muhammad Nur Cahyanto, Mohammad J. Taherzadeh

**Affiliations:** 1 Swedish Centre for Resource Recovery, University of Borås, Allégatan 1, Borås 50190, Sweden; E-Mail: mohammad.taherzadeh@hb.se; 2 Department of Food and Agricultural Product Technology, Faculty of Agricultural Technology, Universitas Gadjah Mada, Bulaksumur, Yogyakarta 55281, Indonesia; E-Mails: ria_millati@ugm.ac.id (R.M.); mcahyanto@yahoo.com (M.N.C.)

**Keywords:** MBR, encapsulation, anaerobic digestion, d-limonene, citrus waste

## Abstract

Rapid acidification and inhibition by d-limonene are major challenges of biogas production from citrus waste. As limonene is a hydrophobic chemical, this challenge was encountered using hydrophilic polyvinylidine difluoride (PVDF) membranes in a biogas reactor. The more sensitive methane-producing archaea were encapsulated in the membranes, while freely suspended digesting bacteria were present in the culture as well. In this membrane bioreactor (MBR), the free digesting bacteria digested the citrus wastes and produced soluble compounds, which could pass through the membrane and converted to biogas by the encapsulated cell. As a control experiment, similar digestions were carried out in bioreactors containing the identical amount of just free cells. The experiments were carried out in thermophilic conditions at 55 °C, and hydraulic retention time of 30 days. The organic loading rate (OLR) was started with 0.3 kg VS/m^3^/day and gradually increased to 3 kg VS/m^3^/day. The results show that at the highest OLR, MBR was successful to produce methane at 0.33 Nm^3^/kg VS, while the traditional free cell reactor reduced its methane production to 0.05 Nm^3^/kg VS. Approximately 73% of the theoretical methane yield was achieved using the membrane bioreactor.

## 1. Introduction

According to Food and Agriculture Organization, orange, as the main citrus fruit, is one of top five fruit commodities in the global fruit market [1]. Global orange production reached 69 million tons in 2012 representing 8.5% of the total fruit production. Approximately 40%–60% of citrus production is processed for juice production, of which 50%–60% ends up as waste [2,3]. The global citrus waste production was 15–25 million tons a year [3]. Having high biodegradability, accumulation of citrus waste creates a serious problem to the environment, such as heavy odor, plenty of leachate, as well as attracting flies and rats [4], thus, a sustainable handling of citrus waste is highly desirable. The most promising alternative to incinerating and composting is converting this waste into biogas via anaerobic digestion [5,6]. Biogas holds several applications such as fuel for vehicles, heating, cooking, and electricity production. In addition, the residue of anaerobic digestion can be used as an excellent soil conditioner after minor treatments [7]. Furthermore, conversion of citrus waste into biogas is a combination of pollutant reduction and energy production.

The biogas production from citrus waste process can be classified into two main steps,* i.e.*, acid formation and methane production. The acid forming and methane forming microorganisms differ widely with regards to physiology, nutritional requirements, growth kinetics, and sensitivity to environmental conditions [8]. Failure to maintain the balance between these two groups leads to instability of the process [9]. Moreover, the doubling time of methanogens (5–15 days) is in order of ten time longer than acid forming bacteria (1–1.5 days) [10]. Consequently, high dilution rate and short retention time result in wash out of methanogens [11]. Encapsulation is an attractive solution to prevent wash out of the methanogen by retaining the cells inside the bioreactor.

Other challenges of anaerobic digestion from citrus waste come from the characteristic of the substrate,* i.e.*, rapid acidification and inhibition caused by limonene. Fruit waste in general is very rapidly acidified into volatile fatty acid (VFA) resulting in low pH and tends to inhibit methane production process [12,13]. Furthermore, a number of studies have reported that presence of limonene in the citrus oil hinders the biogas production of citrus waste [14,15,16]. d-limonene is antimicrobial compound that constitutes 90% of citrus essential peel oil [15]. It causes ultimate failure of the process at concentration of 400 μL/L on mesophilic continuous digestion [15] and in the range of 450 to 900 μL/L on thermophilic batch digestion [14].

Rapid acidification can be overcome by employing two-stage process in two sequential bioreactors for hydrolysis/acidification and methanogenesis. Two-stage system was shown to improve the substrate degradation yield and biogas productivity [13,17,18]. However, two-stage process is less attractive for industry since it requires more complicated design and higher cost for installation and maintenance of the digester [18]. In addition, approximately, 90% of digesters for treatment of organic fraction of municipal solid waste and bio-waste currently in use in Europe are operated in one stage system [19]. Therefore, performing two-stage process in one reactor would be industrially attractive.

Several attempts have been proposed to overcome inhibition problem by limonene, such as pretreatment of the citrus waste to remove the limonene or using the cell protection method [16,20,21]. Citrus waste can be pretreated by several methods including steam explosion [20], steam distillation [16], and acid hydrolysis [22]. However, these methods are performed under harsh conditions, which requires high energy consumption. Hence, cell protection is more favorable in term of energy consumption. Cell protection can be conducted by employing a selective membrane to prevent diffusion of limonene to the cells while still allowing nutrient to pass. d-limonene is a hydrophobic compound, which belongs to terpenoid group. Thus, it is theoretically not permeable to a hydrophilic membrane.

In our previous work [23], encapsulated cell in Polyvinylidene Difluoride (PVDF) membrane was successful to protect the cell from limonene and improve the biogas production using synthetic medium containing cellulose and limonene. A number of microorganisms was added outside the encapsulated cell to digest the cellulose into a soluble compound, which is able to permeate to the membrane and further converted into methane by the encapsulated cell. Having this configuration, the two-stage process can be conducted in one reactor. However, the application of this configuration of membrane bioreactor (MBR) on real fruit waste as solid material has not yet been examined. Furthermore, there is a scarce report in literature for application of MBR on solid waste. MBR is mostly applied in various dilute wastewater treatments such as municipal/domestic wastewater [24], industrial wastewater [25,26], and surface/drinking water [27]. The aim of this work was to evaluate the performance of MBR for improvement of biogas production from citrus waste.

## 2. Results and Discussion

Citrus waste contains 74.53% of carbohydrate, 7.68% of protein, and 10.80% of fat [28]. The limonene content of the citrus waste is 3.78% of the dry weight [29]. Based on the composition, the theoretical methane yield that can be obtained from citrus waste calculated by Equation 1 is 0.45 Nm^3^/kg VS.

C_c_H_h_O_o_N_n_S_s_ + *y*H_2_O → *x*CH_4_ + nNH_3_ + sH_2_S + (c − *x*) CO_2_ (1)

**Figure 1:**
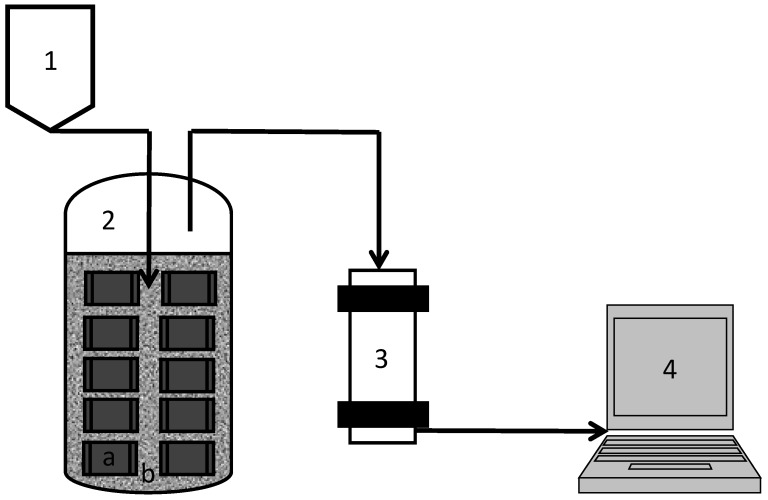
Schematic diagram of semi-continuous digestion using MBR. The numbers indicates (**1**) feeding container containing; (**2**) MBR containing (**a**) encapsulated cells in hydrophilic PVDF membranes and (**b**) free cells; (**3**) gas measuring system; (**4**) controller.

In reality, however, the methane yields of the citrus waste have been reported to be 0.06–0.1 Nm^3^/kg VS [14] which can be explained by the presence of limonene which inhibit the anaerobic digestion process [15]. In this work, MBR was applied to retain the cell in the reactor and to protect the cell from limonene. The configuration of the MBR used in this work is presented in Figure 1. For comparison, a reactor, which contains only free cell, was used as control. The experiment was carried out in semi-continuous reactor and incubated for 83 days. In order to detect the imbalance due to inhibition by limonene, several parameters have been monitored that include gas production, gas composition, pH, and volatile fatty acid profile as stress indicator [30].

### 2.1. Methane Yield and Biogas Composition

**Figure 2:**
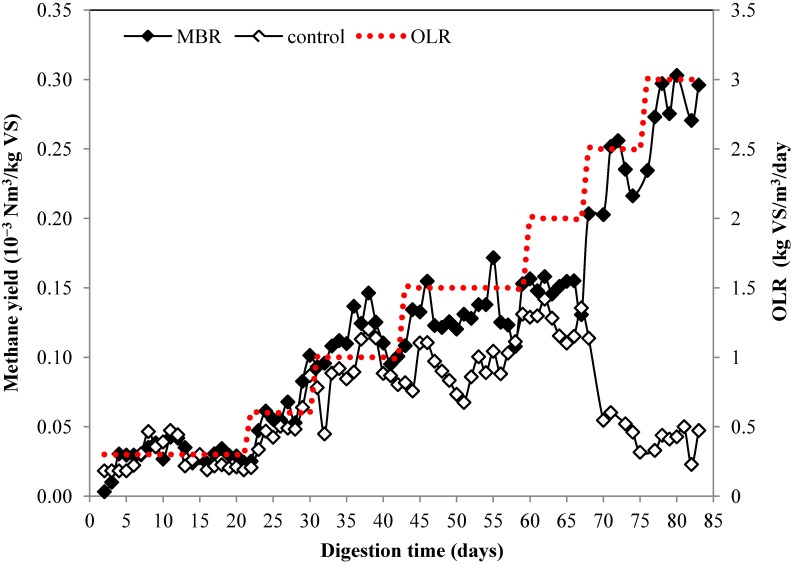
Methane production of anaerobic digestion from citrus waste using MBR and control at different organic loading rate.

The citrus waste sludge was fed to the bioreactor at the loads of 0.3 kg VS/m^3^/day during the startup period. After the methane production was stable, the organic loading rate was gradually increased and reached 3 kg VS/m^3^/day at the end of the digestion. The change of methane production during the digestion process is presented in Figure 2. For MBR, increasing of the organic loading rate (OLR) was followed by increasing of methane production and the maximum methane production was achieved at OLR of 3 kg VS/m^3^/day. The methane production for the control increased up to OLR of 2 kg VS/m^3^/day, which equals to limonene loading of 60 g/m^3^/day. However when the OLR was increased to 2.5 and 3 kg VS/m^3^/day, the methane production started to decrease on day 67. This may be explained by the high loading of limonene, which corresponds to limonene loading of 76 and 91 g/m^3^/day, for OLR of 2.5 and 3 kg VS/m^3^/day, respectively. The limit of limonene oil loading obtained using free cell in this study (60 g/m^3^/day) was slightly higher than that obtained from previous study (55 g/m^3^/day) [15]. This could be due to the higher cell concentration used in this work (240 g/L) compare to that (137.5 g/L) reported by Mizuki *et al.* [15]. In addition, final concentration of limonene at 3.293 kg/m^3^ did not affect the performance of MBR in this study, whereas final concentration of limonene at 378–756 g/m^3^ has been reported to lead to process failure [14]. The inhibitory mechanism of limonene on anaerobic digestion is unclear. However, the toxicity of limonene on *Saccharomyces cerevisiae* have been suggested due to membrane cellular disruption resulting in leak out of the cells components and disturbance of H^+^ and K^+^ transport energized by glycolysis [31].

**Figure 3:**
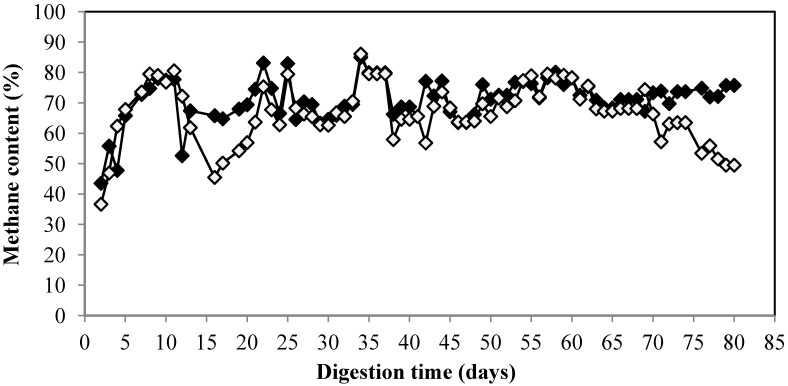
Methane content of biogas from citrus waste using MBR (**♦**) and control (**◊**) during digestion.

The methane yield for control started to decrease from 0.3 Nm^3^/kg VS at OLR of 1 kg VS/m^3^/day to 0.21 Nm^3^/kg VS at OLR of 1.5 kg VS/m^3^/day. The methane yield was only 0.05 Nm^3^/kg VS at the highest OLR at 3 kg VS/m^3^/day corresponding to 11% from theoretical methane yield (Table 1). On the contrary, the methane yield of MBR remained high on high OLR. The methane yield of MBR in various OLR was 0.25–0.38 Nm^3^/kg VS which corresponded to 55%–84% from theoretical yield. The methane yield at the highest OLR, 3 kg VS/m^3^/day, was 0.33 Nm^3^/kg VS corresponding to 73% from the theoretical yield. The results showed that MBR could improve the methane yield by more than six times from 0.05 to 0.33 Nm^3^/kg VS at the highest OLR and this emphasizes the benefit of MBR over the conventional free cell. Besides improvement of the methane yield, the encapsulation offers other advantage such as easier cell recovery from the bioreactor in downstream process [32]. Moreover, this technique offers other advantage by protection of methanogen from harsh environmental condition such as change of pH, temperature, and accumulation of short chain organic acid which negatively affect the stability of the whole process [33]. In addition, no energy is required for pretreatment. The biogas composition of the MBR remained stable with the methane content was about 79%. On the contrary, the methane content for the control decreased and only 49% of methane was produced in the end of experiment (Figure 3).

**Table 1:** Methane yield of anaerobic digestion from citrus waste using membrane bioreactor (MBR) and free cell at different organic loading rate.

OLR (kg VS/m^3^/day)	Methane Yield (Nm^3^/kg VS)		Percentage from Theoretical Yield (%) *	
	Control	MBR	Control	MBR
0.3	0.3	0.32	67	71
0.6	0.27	0.34	60	75
1	0.3	0.38	67	84
1.5	0.21	0.29	47	64
2	0.21	0.25	47	56
2.5	0.1	0.32	22	71
3	0.05	0.33	11	73

* Theoretical methane yield of citrus waste is 0.45 Nm^3^/kg VS.

#### 2.1.1. Volatile Fatty Acids Composition

Anaerobic digestion involved four different groups of microorganism namely hydrolytic bacteria, acidogens, acetogens, and methanogens with mutual dependence one to each other, particularly for energetic reason. The hydrolytic bacteria play a role in the first step by degrading complex organic matters into their monomers such as sugars, amino acids and fatty acids. The soluble monomers were then converted into short chain organic acid, acetic acid, alcohols, hydrogen and carbondioxide by acidogen. The acidogenesis products were then subsequently converted into acetic acid by acetogens and finally converted into methane by methanogen. VFA has been suggested for a long time as one of the most important parameters in depicting the anaerobic digestion process [34,35,36]. Therefore, in this work VFA including acetic acid, propionic acid, butyric acid, isobutyric acid, valeric acid, isovaleric acid, and caproic acid were analyzed.

**Figure 4:**
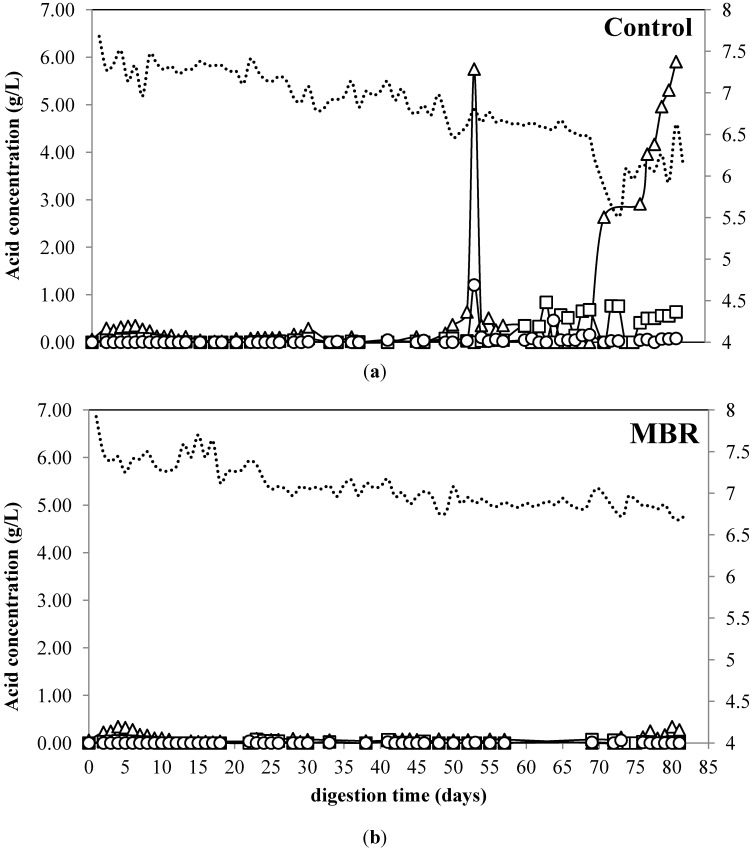
Acetic acid (**∆**), propionic acid (**□**), isobutyric acid (**○**), and pH (**---**) of anaerobic digestion from citrus waste using (**a**) MBR and (**b**) control.

The VFA profiles and pH changes of citrus waste digestion using MBR and free cell are presented in Figure 4. No accumulation of VFA was observed for MBR during the digestion. The concentration of valeric, butyric, and caproic acid did not change during the digestion for both MBR and control (data not shown). For control, no accumulation of VFA was observed during the first 55. However, as the OLR was increased from 1.5 to 2 kg VS/m^3^/day, the acetic acid sharply increased by almost ten times from 0.64 to 5.75 g/L on day 60 and remained high until the end of digestion. Similarly, the isobutyric acid started to accumulate and reached 1.2 g/L. The accumulation of VFA indicates the inhibition occurred for the methanogen, since the VFA produced by acidogens and acetogens was not converted into methane.

VFA is not only an indicator of process imbalance, but it also can inhibit the microorganism at certain levels. For instance, glucose fermentation is inhibited at VFA concentrations above 4 g/L [37]. Since the VFA concentration for the control reactor was above the inhibitory concentration of VFA, the inhibition of the process was not only caused by limonene but also the VFA. Based on the membrane structure, cell membrane of methanogen is made from ether lipids, which lack l-muramic acid and this produces fatty acid-sensitive cell [10]. The methane production and pH remained stable during the accumulation of VFA. This is in accordance with previous study reported that gas production, gas composition, and pH are often too slow to detect a sudden change in the system [38]. The MBR, on the contrary, no accumulation was observed during the digestion process which underlines no inhibition occurred in this system.

The membrane used in this work is hydrophilic membrane, thus theoretically it allows nutrient to pass while retaining the limonene outside the membrane. The high methane yield obtained from MBR compared to the theoretical yield is a proof that there was no problem with mass transfer of the nutrients. Meanwhile, the higher methane production of MBR than that of control supports the fact that the membrane could protect the cell from limonene. Methanogenesis is often considered as a rate limiting step for biogas production from cellulose-poor substrate, such as fruit waste [39]. Hence, protection of the cells particularly for methanogen is a major route to improve the performance of anaerobic digesters [40]. The configuration of MBR in this work enabled to separate the acid forming process outside the membrane and methane forming which occurred inside the membrane. Hence, the MBR behaved as a two-stage system although it was carried out in a single reactor. In large scale, the membrane layer can be inserted in the middle of the reactor to separate acid forming and methane forming process.

## 3. Experimental Section

### 3.1. Inoculum

Inoculum was a sludge obtained from a 3000 m^3^ thermophilic biogas plant (55 °C) at Borås Energy and Environment AB (Borås, Sweden). The sludge was stored in incubator at temperature 55 °C for 2–3 days to acclimate the bacteria with the operation condition. The sludge was stirred in order to obtain homogenous inoculum and the any remaining large particle was removed by passing the sludge through a sieve with a pore size of 1 mm. The sludge was then centrifuged at 14,000 rpm for 10 min and the supernatant was discarded. The pellet was further used as inoculums.

### 3.2. Citrus Waste

The citrus waste used in this work was a residue of orange obtained from Brämhult juice factory (Borås, Sweden) and stored at −20 °C until use. The citrus waste was thawed and grounded to obtain citrus waste slurry. A basal medium was added to the slurry. The composition of basal medium was according to a method previously described [41] with minor modification containing (mg/L): NH_4_Cl (1200); MgSO_4_·7H_2_O (400): KCl (400); CaCl_2_·2H_2_O (50); (NH_4_)_2_HPO_4_ (80); FeCl_2_·4H_2_O (40); CoCl_2_·2H_2_O (10); KI (10); MnCl_2_·4H_2_O (0.5); CuCl_2_·2H_2_O (0.5); ZnCl_2_ (0.5); AlCl_3_·6H_2_O; Na_2_MoO_4_·2H_2_O (0.5); H_3_BO_3_ (0.5); NiCl_2_·6H_2_O (0.5); Na_2_WO_4_·2H_2_O (0.5); Na_2_SeO_3_ (0.5); cysteine (10). The pH of the basal medium was adjusted to 7 ± 0.5 by adding 2 M NaOH.

### 3.3. Preparation of Membrane Encapsulated Cells

The encapsulated cell was prepared by inserting three grams of the pellet into a sachet made from PVDF (Durapore^®^, Thermo Fisher Scientific, Inc., Göteborg, Sweden) as previously reported [11,41]. The properties of PVDF used in this work were: hydrophilic with water flow rate of 2.5 mL/min × cm^2^; air flow rate of 0.15 mL/min × cm^2^; pore size of 0.1 µm; thickness of 125 µm; porosity of 70%; and maximum operating temperature of 85 °C. The membrane sachet was prepared by cutting the membrane into size of 3 cm × 6 cm, and sealed on two sides of the membrane using a sealer (HPL 450 AS, Hawo, Mosbach, Germany) with heating and cooling time of 4.5 s of each. After the insertion of the cells, the sachet was sealed to close. The membrane-encapsulated cells were immediately used for the experiment.

### 3.4. Semi-Continuous Digestion

The anaerobic digestion was carried out in semi-continuous 500 mL glass bottle reactors. The reactor was incubated in waterbath for 75 days at 55 °C. For membrane bioreactor, a total of 72 g of inoculums was used in the form of free cells and membrane encapsulated cells with ratio of 1:1. The free cells were prepared by dissolving 36 g of pellets into 300 mL of distilled water and placed to the reactor. Twelve sachets of encapsulated cell each containing 3 g of pellet were placed in a basket and immersed in the reactor. For control reactor, 72 g of pellet was dissolved in 300 mL of distilled water. The organic loading rate was set at 0.3 kg VS/m^3^/day during start-up period. The OLR was then gradually increased to 3 kg VS/m^3^/day. The hydraulic retention time was 30 days. The reactors were sealed with rubber stoppers with two holes, one for feeding and sampling (inlet) and the other one for collecting and measuring the gas (outlet). All reactors were shaken twice a day. Samples were withdrawn every day for analysis and the pH was measured from the samples immediately.

### 3.5. Analytical Method

Gas production from the outlet of the reactor was automatically monitored using a data acquisition system (AMPTS II, Bioprocess control, Sweden AB, Lund, Sweden). Gas composition was analysed using a gas chromatograph (Clarus 500, Perkin-Elmer, Waltham, MA, USA) equipped with a packed column (Perkin Elmer, 6ʹ × 1.8ʺ OD, 80/100, Mesh, USA) and a thermal conductivity detector (Perkin Elmer, Waltham, MA, USA) with inject temperature of 150 °C. The carrier gas was nitrogen operated with a flow rate of 20 mL/min at 60 °C. Gas sampling was conducted using a 0.25 µL (VICI, Baton Rouge, LA, USA).

Volatile fatty acids were determined using GC (Clarus 500, Perkin-Elmer, Waltham, MA, USA) on a Flame Ionized Detector (FID) equipped with Capillary column (Zebron ZB-WAXplus, Phenomenex, Torrance, CA, USA). The temperatures of the injector and detector were 250 °C and 300 °C, respectively. Nitrogen at 2 mL/min and pressure of 20 °C was used as a carrier gas. The oven temperature was initially 50 °C and gradually increased to 200 °C at the rate of 40 °C/min and maintained at this temperature for 3.5 min. Autosampler (Clarus 500, Perkin-Elmer, Waltham, MA, USA) was employed for sampling with injection size of 1.5 µL and split was set at 15:1.

## 4. Conclusions

Application of membrane bioreactor on citrus waste successfully improved the biogas production by more than six times compared to the conventional digestion system and reached 73% of theoretical methane yield. This underlines the potential application of MBR for citrus waste and other solid wastes containing hydrophobic inhibitors.
